# Increased Risk of Peptic Ulcers Following a Cholecystectomy for Gallstones

**DOI:** 10.1038/srep30702

**Published:** 2016-07-29

**Authors:** Ming-Chieh Tsai, Chung-Chien Huang, Li-Ting Kao, Herng-Ching Lin, Cha-Ze Lee

**Affiliations:** 1 Division of Gastroenterology, Department of Internal Medicine, Hsinchu Cathay General Hospital, Taipei, Taiwan; 2 School of Health Care Administration, Taipei Medical University, Taipei, Taiwan; 3 Graduate Institute of Life Science, National Defense Medical Center, Taipei, Taiwan; 4 Sleep Research Center, Taipei Medical University Hospital, Taipei, Taiwan; 5 Department of Internal Medicine, National Taiwan University Hospital and College of Medicine, Taipei, Taiwan

## Abstract

This retrospective cohort study examined the relationship between a cholecystectomy and the subsequent risk of peptic ulcers using a population-based database. Data for this study were retrieved from the Taiwan Longitudinal Health Insurance Database 2005. This study included 5209 patients who had undergone a cholecystectomy for gallstones and 15,627 sex- and age-matched comparison patients. We individually tracked each patient for a 5-year period to identify those who subsequently received a diagnosis of peptic ulcers. We found that of the 20,836 sampled patients, 2033 patients (9.76%) received a diagnosis of peptic ulcers during the 5-year follow-up period: 674 from the study group (12.94% of the patients who underwent a cholecystectomy) and 1359 from the comparison group (8.70% of the comparison patients). The stratified Cox proportional hazard regressions showed that the adjusted hazard ratio (HR) for peptic ulcers during the 5-year follow-up period was 1.48 (95% CI = 1.34~1.64) for patients who underwent a cholecystectomy than comparison patients. Furthermore, the adjusted HRs of gastric ulcers and duodenal ulcers during the 5-year follow-up period were 1.70 and 1.71, respectively, for patients who underwent a cholecystectomy compared to comparison patients. This study demonstrated a relationship between a cholecystectomy and a subsequent diagnosis of peptic ulcers.

A cholecystectomy is the gold standard treatment for symptomatic gallstones^1^,^2^. However, some studies reveal that after a cholecystectomy, about 7~47% of patients are dissatisfied with the procedure; the most common reason for this is related to postcholecystectomy syndrome (PCS)^1^,^2^,^3^. Previous studies reported that duodenogastric reflux (DGR) with bile reflux gastritis (BRG) following a cholecystectomy may contribute to some persistence of PCS^4^,^5^,^6^,^7^,^8^,^9^,^10^,^11^,^12^,^13^,^14^,^15^.

DGR is a natural physiological phenomenon attributed to the reflux of duodenal contents from the duodenum into to the stomach which presents for a short period and causes few symptoms^16^,^17^. However, the pathological DGR is very common in adults after a cholecystectomy^4^,^5^,^6^,^7^,^8^,^9^,^17^. Previous reports showed that 20~85% of post-cholecystectomy symptomatic patients have pathological DGR within 6 months after a cholecystectomy^8^,^11^,^12^,^13^,^14^. Pathological DGR occurs when the reflux of pancreatic and biliary secretions is excessive or lasts for a long time and subsequently damages the gastric mucosa and leads to the development of BRG and peptic ulcers^15^,^18^,^19^,^20^,^21^. Furthermore, studies demonstrated that DGR may suppress the somatostatin concentration that is produced by the alkaline nature or pancreatic component of the reflux, which subsequently results in hypersecretion of acid and hypergastrinemia^18^,^22^,^23^,^24^. DGR may therefore be considered a common factor in the pathogenesis of gastric and duodenal ulcers^23^,^24^,^25^,^26^.

However, all such studies relied upon regional samples, or on data from a few selected hospitals or subpopulations of patients, and as such, do not permit unequivocal conclusions. In addition, according to our knowledge, no study has attempted to investigate the risk of peptic ulcers following a cholecystectomy. Therefore, this retrospective cohort study aimed to examine the relationship between a cholecystectomy and the subsequent risk of peptic ulcers using a population-based database.

## Methods

### Database

Data for this retrospective cohort study were retrieved from the Longitudinal Health Insurance Database 2005 (LHID2005). The LHID2000, which was created and released by the Taiwan National Health Research Institute (NHRI), includes medical claims of 1,000,000 enrollees. These 1,000,000 enrollees were randomly selected by the Taiwan NHRI from enrollees listed in the 2005 Registry of Beneficiaries (*n* = 27,378,403) under the Taiwan National Health Insurance (NHI) program. The LHID2005 provides an exclusive opportunity for researchers in Taiwan to longitudinally explore the relationship between a cholecystectomy and the risk of peptic ulcers.

This study was exempted from full review by the Institutional Review Board of the National Defense Medical Center because the LHID2005 consists of de-identified secondary data released to the public for research purposes.

### Study sample

This study was designed to include a study group and a comparison group. We selected the study group by identifying 6834 patients who had undergone a cholecystectomy (International Classification of Diseases, 9th edition, Clinical Modification (ICD-9-CM) procedure codes 51.2, 51.21, 51.22, 51.23, and 51.24) during January 1, 2002 to December 31, 2008. We also assured that all included patients had received a discharge diagnosis of gallstones (ICD-9-CM codes 574.0~574.4, and 574.6~574.9). In Taiwan, all gallstones diagnoses were confirmed using an ultrasound scan. In addition, we excluded 11 patients aged less than 18 years in order to limit the study sample to the adult population. We defined the date of the cholecystectomy as the index date for the study group. Thereafter, we excluded 1614 patients who had ever received a diagnosis of peptic ulcers within 3 years prior to the index date. As a result, the study group included 5209 patients who had undergone a cholecystectomy for gallstones.

Similarly, we retrieved the comparison group from the remaining enrollees in the Registry of Beneficiaries of the LHID2005. We first excluded enrollees who had a history of gallstones or who had undergone a cholecystectomy. However, the LHID2005 did not allow us to identify and exclude patients who had undergone a cholecystectomy prior to 1995 because the NHI program began in 1995. This potential bias would lead the findings of this study toward the null hypothesis. We subsequently used the SAS procsurveyselect program (SAS System for Windows, vers. 8.2, SAS Institute, Cary, NC, USA) to randomly select 15,627 subjects (3 for every patient who underwent a cholecystectomy) matched according to sex, age group, and the year of the index date. For the comparison group, the year of the index date was simply a matched year during which the patients used healthcare services. We then assigned their first healthcare use occurring in the index year as their index date for the comparison group. Moreover, we ensured that all selected comparison patients did not undergo a cholecystectomy during the 5-year follow-up period. We also assured that none of the selected comparison patients had a history of peptic ulcers within 3 years prior to the index date.

Ultimately, this study included 20,836 sampled patients. We individually tracked each patient for 1, 3, and 5 years starting from the index date to identify those who subsequently received a diagnosis of peptic ulcers (ICD-9-CM codes 531~533) in ambulatory care visits (including outpatient departments of hospitals and clinics) during the follow-up period. In Taiwan, when a gastroenterologist suspects that a patient has a peptic ulcer, he/she may provide the patient with a temporary diagnosis of a peptic ulcer in order to perform the associated clinical examinations, endoscopies or lab tests for confirmation without receiving a monetary penalty from the Bureau of the NHI. The peptic ulcer commonly diagnosed by gastroenterologists based upon characteristic symptoms and endoscopies. If a patient has a confirmed peptic ulcer diagnosis after an examination, the patient will receive routine therapy and have a second peptic ulcer diagnosis in their next outpatient visit. Therefore, in order to increase the validity of the diagnoses of peptic ulcers, we only selected peptic ulcer cases if they received two or more peptic ulcer diagnoses.

### Statistical analysis

The SAS system was used for statistical analyses in this study. We used Pearson Chi-squared tests to compare differences in medical comorbidities between patients who underwent a cholecystectomy and comparison patients. These comorbidities included hypertension (ICD-9-CM codes 401-405), hyperlipidemia (ICD-9-CM codes 272.0-272.4), diabetes (ICD-9-CM codes 250), coronary heart disease (CHD) (ICD-9-CM codes 410-414 or 429.2), obesity (ICD-9-CM codes 278, 278.0, or 278.00-278.01), alcohol abuse/alcohol dependence syndrome (ICD-9-CM codes 303), and tobacco use disorder (ICD-9-CM codes 305.1). We only counted comorbidities if the condition occurred in an inpatient setting or appeared in two or more ambulatory care claims coded before the index date in this study. Furthermore, we used stratified Cox proportional hazard regressions (stratified by age group, sex, and the year of the index date) to compute hazard ratios (HRs) and corresponding 95% confidence intervals (CIs) for peptic ulcers during the 5-year follow-up period in patients who underwent a cholecystectomy and comparison patients, with cases censored if individuals died during that time (839 [16.1%] from the study cohort and 1755 [12.3%] from the comparison cohort). The significance level was *p* = 0.05 for this study.

## Results

Table 1 presents the distributions of demographic characteristics and medical comorbidities stratified by the presence or absence of a cholecystectomy. After the patients were matched according to sex, age group, and the year of the index date, we found that patients who underwent a cholecystectomy were more likely to have the comorbidities of hypertension, hyperlipidemia, diabetes, CHD, obesity, alcohol abuse/alcohol dependence syndrome, and tobacco use disorder (all *p* < 0.001) than were comparison patients.

Table 2 presents the incidence of peptic ulcers during the follow-up period. Of the 20,836 sampled patients, 2033 patients (9.76%) received a diagnosis of peptic ulcers during the 5-year follow-up period: 674 from the study group (12.94% of the patients who underwent a cholecystectomy) and 1359 from the comparison group (8.70% of comparison patients) (*p* < 0.001).

Table 2 also presents the crude and adjusted HRs for peptic ulcers. The stratified Cox proportional hazard regressions (stratified by age group, sex, and the year of the index date) showed that the HRs for peptic ulcers during the 1-, 3-, and 5-year follow-up periods were 2.34 (95% CI = 1.98~2.77), 1.84 (95% CI = 1.63~2.06), and 1.56 (95% CI = 1.41~1.72), respectively, for patients who underwent a cholecystectomy compared to comparison patients. Furthermore, the HR of peptic ulcers during the 5-year follow-up period was 1.48 (95% CI: 1.34~1.64) for patients who underwent a cholecystectomy after censoring individuals who died during the follow-up period and adjusting for the patients’ geographic location, hypertension, hyperlipidemia, diabetes, CHD, obesity, alcohol abuse/alcohol dependence syndrome, and tobacco use disorder.

Table 3 presents the crude and adjusted HRs for gastric ulcers and duodenal ulcers. We found that the adjusted HRs of gastric ulcers and duodenal ulcers during the 5-year follow-up period were 1.70 (95% CI: 1.44~1.99) and 1.71 (95% CI: 1.36~2.15), respectively, for patients who underwent a cholecystectomy compared to comparison patients. Patients who underwent a cholecystectomy had an increased risk of peptic ulcers regardless of the type of peptic ulcer.

## Discussion

This retrospective cohort study found that 12.94% of the patients who underwent a cholecystectomy had peptic ulcers during 5-year follow-up period compared to 8.70% of patients who did not undergo a cholecystectomy. The Cox proportional hazard regression analysis suggested that a cholecystectomy was significantly associated with an increased risk of subsequent peptic ulcers (adjusted HR = 1.48). In addition, the adjusted HRs of gastric ulcers and duodenal ulcers during the 5-year follow-up period were 1.70 and 1.71, respectively, for patients who underwent a cholecystectomy compared to comparison patients.

The current findings on the incidence rates of peptic ulcers were parallel to those of prior studies, all of which reported that 3.0~10% of patients develop peptic ulcers following a cholecystectomy^15^,^27^,^28^,^29^. The mechanisms underlying the relationship between a cholecystectomy and peptic ulcers remain unclear. One possible explanation could be that DRG, which damages the gastric mucosa by bile acids and pancreatic phospholipase A2, leads to peptic ulcers^20^,^21^. In addition, some animal studies demonstrated that DGR suppresses somatostatin concentrations and increases intragastric gastrin and acid secretion^18^,^22^,^23^,^24^,^25^,^26^. Therefore, DGR may be considered a factor in the pathogenesis of gastric and duodenal ulcers as suggested by some researchers^18^,^30^,^31^. Furthermore, previous studies indicated that antroduodenal motility is altered and gastric emptying is delayed after a cholecystectomy, which may also play a possible role in the etiology of gastric ulcers^32^,^33^.

In addition to gastric acid and chemical injury by bile acids related to peptic ulcers, *Helicobacter pylori* is considered to play the most important role in peptic ulcers nowadays^34^,^35^. Recent studies suggested that *H. pylori* infection is one possible cause of gallstones^30^,^31^,^36^. The synergistic effect of a high reflux bile acid load and *H. pylori* infection is likely to be a critical event in the pathogenesis of peptic ulcers^37^,^38^.

The present study showed that the HRs for peptic ulcers during the 1-, 3-, and 5-year follow-up periods decreased with time, and were 2.34, 1.84, and 1.56, respectively, for patients who underwent a cholecystectomy compared to comparison patients. Previous studies suggested that the healing and regeneration of the gastric mucosa against repeated bile damage from chronic DRG could result in chronic atrophic gastritis and intestinal metaplasia^39^,^40^. Wilson *et al.* also reported that prolonged DRG in patients of longer than 3 years after a cholecystectomy caused the gastric pH to exceed 3 an increased percentage of the time and was associated with a high incidence of chronic gastritis^9^. Therefore, according to morphological and physiological changes of the gastric mucosa with chronic excess DRG, the incidence of peptic ulcers could decrease with time due to atrophic gastric mucosa with hypochlorhydria.

This study has several strengths. First, a population-based dataset with a large sample size was used to explore the relationship between a cholecystectomy and peptic ulcers. The large sample size afforded a considerable statistical advantage in detecting actual differences between the study group and comparison group. Second, the diagnosis of peptic ulcers by certified gastroenterologists has a very high validity since it is diagnosed by panendoscopy in Taiwan. In addition, this study only included peptic ulcer cases if they had received two or more peptic ulcer diagnoses.

Nevertheless, the results of this study need to be seen in the light of several limitations. First, the LHID 2005 provides no information on the histology or existence of *H. pylori* in gastric biopsies. In addition, this database has no relevant records on data for laboratory examination, biochemical examination, or diagnostic imaging and so it was not possible to differentiate between indications for cholecystectomy (biliary colic, cholecystitis or pancreatitis). Second, physicians do not routinely biopsy the gastric mucosa to identify BRG in Taiwan. This may prevent researchers from identifying the pathogenesis of cholecystitis and ERG. Third, although we took important comorbidities (e.g., hypertension, diabetes, and alcohol abuse) into consideration in the analysis, it is still possible that patients who underwent a cholecystectomy might possess higher risks of peptic ulcers due to undetectable factors in our claims dataset (e.g., a genetic tendency, poor lifestyle, or other illnesses). Finally, there may have been a surveillance bias in that patients who underwent a cholecystectomy were more likely to have frequent outpatient clinic visits, which may have led to early detection of peptic ulcers.

Despite the aforementioned limitations, this study demonstrated a relationship between a cholecystectomy and a subsequent diagnosis of peptic ulcers. In patients who underwent a cholecystectomy, peptic ulcers contribute to some symptoms of PCS. We suggest that clinicians be alert and suspect peptic ulcers with persistent epigastric pain or dyspepsia in patients with a prior cholecystectomy. In addition, future studies need to elucidate the mechanisms underpinning the relationship detected in this study.

## Additional Information

**How to cite this article**: Tsai, M.-C. *et al.* Increased Risk of Peptic Ulcers Following a Cholecystectomy for Gallstones. *Sci. Rep.* **6**, 30702; doi: 10.1038/srep30702 (2016).

## Figures and Tables

**Table 1:** Demographic characteristics of sampled patients (N = 20,836).

Variable	Patients who underwent a cholecystectomy *N* = 5209		Comparison patients *N* = 15,627		*p* value
	Total no.	Column %	Total no.	Column %	
Male	2429	59.1	3825	59.1	>0.999
Age group (years)					>0.999
18~29	220	4.2	660	4.2	
30~39	660	12.7	1980	12.7	
40~49	936	18.0	2808	18.0	
50~59	1159	22.2	3475	22.2	
60~69	931	17.9	2793	17.9	
>69	1303	25.0	3909	25.0	
Geographic region					<0.001
Northern	2490	47.8	7260	46.5	
Central	1058	20.3	3765	24.1	
Southern	1524	29.3	4229	27.1	
Eastern	137	2.6	373	2.4	
Diabetes	1326	25.5	2638	16.9	<0.001
Hypertension	2559	49.1	5850	37.4	<0.001
Coronary heart disease	1181	22.7	2355	15.1	<0.001
Hyperlipidemia	1741	33.4	4111	26.3	<0.001
Obesity	100	1.9	156	1.0	<0.001
Alcohol abuse/alcohol dependence syndrome	43	0.8	52	0.3	<0.001
Tobacco use disorder	248	4.8	288	1.8	<0.001

**Table 2:** Incidences, hazard ratios (HRs), and 95% confidence intervals (CIs) for peptic ulcers among sampled patients.

Presence of peptic ulcers	Total sample *N* = 20,836		Patients who underwent a cholecystectomy *N* = 5209		Comparison patients *N* = 14,268	
1-year follow-up period						
Yes, *n* (%)	563	2.70	243	4.67	320	2.05
HR (95% CI)	—		2.34*** (1.98~2.77)		1.00	
Adjusted HR^a^ (95% CI)	—		2.21*** (1.86~2.63)		1.00	
3-year follow-up period						
Yes, *n* (%)	1299	6.23	480	9.21	819	5.24
HR (95% CI)	—		1.84*** (1.63~2.06)		1.00	
Adjusted HR^a^ (95% CI)	—		1.73*** (1.54~1.95)		1.00	
5-year follow-up period						
Yes, *n* (%)	2033	9.76	674	12.94	1359	8.70
HR (95% CI)	—		1.56*** (1.41~1.72)		1.00	
Adjusted HR^a^ (95% CI)	—		1.48*** (1.34~1.64)		1.00	

HRs were calculated by Cox proportional hazard regressions stratified by age group, sex, and the year of the index date; ^a^After adjusting for the patients’ geographic region, hypertension, hyperlipidemia, diabetes, coronary heart disease, obesity, alcohol abuse/alcohol dependence syndrome, and tobacco use disorder.***p < 0.001.

**Table 3:** Incidences, hazard ratios (HRs), and 95% confidence intervals (CIs) for gastric ulcers and duodenal ulcers among sampled patients during a 5-year follow-up period.

Outcomes	Patients who underwent a cholecystectomy *n* = 5209		Comparison patients *n* = 14,268	
	No.	%	No.	%
5-year follow-up period				
Presence of gastric ulcers	249	4.78	426	2.73
HR (95% CI)	1.79*** (1.53~2.10)		1.00	
Adjusted HR^a^ (95% CI)	1.70*** (1.44~1.99)		1.00	
Presence of duodenal ulcers	125	2.40	208	1.33
HR (95% CI)	1.82*** (1.46~2.28)		1.00	
Adjusted HR^a^ (95% CI)	1.71*** (1.36~2.15)		1.00	

HRs were calculated by Cox proportional hazard regressions stratified by age group, sex, and the year of the index date; ^a^After adjusting for the patients’ geographic region, hypertension, hyperlipidemia, diabetes, coronary heart disease, obesity, alcohol abuse/alcohol dependence syndrome, and tobacco use disorder. ***p < 0.001.
